# Supplementary material for: Mechanisms governing the pioneering and redistribution capabilities of the non-classical pioneer PU.1
Source: Nat Commun. 2020 Jan 21;11:402. doi: 10.1038/s41467-019-13960-2 (PMC6972792; doi:10.1038/s41467-019-13960-2)
Supplement: Supplementary file 7 — Source data [file 41467_2019_13960_MOESM7_ESM.zip › Source_Data/FigureS5/FigureS5B_MotifsETS1specificPeaks/homerResults/motif27.similar.html]

motif27

## Information for motif27

C
G
A
T
A
T
G
C
A
C
G
T
A
C
G
T
A
C
G
T
A
T
G
C
A
G
T
C
A
T
G
C
A
C
G
T
A
T
C
G
C
T
G
A
A
C
T
G
  
Reverse Opposite:  

A
G
T
C
A
G
C
T
T
A
G
C
C
G
T
A
T
A
C
G
A
C
T
G
A
T
C
G
C
G
T
A
C
G
T
A
C
G
T
A
A
T
C
G
C
G
T
A
  

|  |  |
| --- | --- |
| p-value: | 1e-13 |
| log p-value: | -3.114e+01 |
| Information Content per bp: | 1.823 |
| Number of Target Sequences with motif | 18.0 |
| Percentage of Target Sequences with motif | 1.59% |
| Number of Background Sequences with motif | 4.0 |
| Percentage of Background Sequences with motif | 0.05% |
| Average Position of motif in Targets | 99.8 +/- 58.0bp |
| Average Position of motif in Background | 80.2 +/- 42.3bp |
| Strand Bias (log2 ratio + to - strand density) | 0.2 |
| Multiplicity (# of sites on avg that occur together) | 1.00 |
| Motif File: | file (matrix) reverse opposite |

### Similar de novo motifs found

|  |  |  |  |  |  |  |  |
| --- | --- | --- | --- | --- | --- | --- | --- |
| Rank | Match Score | Redundant Motif | P-value | log P-value | % of Targets | % of Background | Motif file |
| 1 | 0.892 | T C G A A G C T A T G C G A C T A G C T G A C T T A G C A G T C A G T C G A C T A T G C C G T A A T C G | 1e-12 | -28.569697 | 2.11% | 0.19% | motif file (matrix) |
